# Age- and sex-associated differences in hematology and biochemistry parameters of Dunkin Hartley guinea pigs (*Cavia porcellus*)

**DOI:** 10.1371/journal.pone.0253794

**Published:** 2021-07-09

**Authors:** Alexa P. Spittler, Maryam F. Afzali, Sydney B. Bork, Lindsey H. Burton, Lauren B. Radakovich, Cassie A. Seebart, A. Russell Moore, Kelly S. Santangelo

**Affiliations:** Department of Microbiology, Immunology, & Pathology, College of Veterinary Medicine and Biomedical Sciences, Colorado State University, Fort Collins, Colorado, United States of America; Federal University of Western Pará, BRAZIL

## Abstract

The Dunkin Hartley is the most common guinea pig strain used in biomedical research, particularly for studies of asthma, allergy, infectious disease, reproduction, and osteoarthritis. Minimally invasive blood tests, such as complete blood counts and serum biochemistry profiles, are often collected for diagnostics and laboratory analyses. However, reference intervals for these assays have not yet been well-documented in this strain. The purpose of this study was to establish reference intervals for hematologic and biochemical parameters of Dunkin Hartley guinea pigs and determine age- and sex-related differences. Hematologic and biochemical parameters were retrospectively obtained from 145 male and 68 female guinea pigs between 2 and 15 months of age. All blood parameters were analyzed by a veterinary clinical pathology laboratory. Reference intervals were established according to the American Society for Veterinary Clinical Pathology guidelines. Age- and sex-related differences were determined using unpaired t-tests or nonparametric Mann-Whitney tests. Hematocrit, red blood cell distribution width, mean platelet volume, white blood cell count, heterophils, monocytes, eosinophils, glucose, blood urea nitrogen, creatinine, calcium, magnesium, total protein, albumin, globulin, cholesterol, aspartate aminotransferase, gamma glutamyl transferase, and bicarbonate increased with age. Mean corpuscular hemoglobin concentration, cellular hemoglobin concentration mean, platelets, lymphocytes, phosphorus, albumin/globulin ratio, alkaline phosphatase, anion gap, and calculated osmolality decreased with age. Males had higher hemoglobin, hematocrit, red blood cell count, mean corpuscular hemoglobin concentration, white blood cell count, heterophils, Foa-Kurloff cells, alanine aminotransferase, and bicarbonate and lower mean corpuscular volume, red blood cell distribution width, platelets, mean platelet volume, eosinophils, total protein, albumin, globulin, cholesterol, potassium, anion gap, calculated osmolality, and iron compared to females. Establishing age and sex differences in hematologic and biochemical parameters of Dunkin Hartley guinea pigs provides valuable insight into their physiology to better evaluate diagnostics and experimental results.

## Introduction

Due to their docile nature, small size, and biological similarities to humans, guinea pigs (*Cavia porcellus*) have been a mainstay of biomedical research for hundreds of years. They are most commonly used in allergy, immunology, infectious disease, nutritional, auditory, and osteoarthritis studies, among others [1,2]. The standard laboratory guinea pig for research is the Dunkin Hartley, an outbred, smooth-coated, albino strain. This strain was first developed by Dunkin and Hartley in 1926 and is commercially available from several laboratory breeders [1].

Complete blood counts (CBC) and serum biochemistries are routine blood tests performed to screen health status in both animals and humans. Reference intervals, defined as the set of values comprising 95% of the healthy reference population [3], are essential for laboratory diagnostic testing, as well as clinical decision-making. The American Society for Veterinary Clinical Pathology (ASVCP) has set forth guidelines for determining reference intervals for veterinary species [3]. Despite their widespread use in research, there are few publications reporting hematology and clinical chemistry reference intervals in clinically healthy guinea pigs, particularly of the Dunkin Hartley strain. Waner et al. compared hematologic and clinical chemistry parameters of 1 month old, male haired (n = 10) and hairless (n = 12) Dunkin Hartley guinea pigs [4], but did not include a high enough sample size to establish reference intervals as put forth by the ASVCP guidelines [3]. Prior studies have determined reference intervals in Weiser-Maples [5] and strain 13 [6] guinea pigs. However, there are likely differences in hematology and biochemistry parameters between strains of guinea pigs, similar to other laboratory rodents [7–9]. These studies in guinea pigs [5,6] and other laboratory rodents [8–12] have also demonstrated age and sex to be important factors impacting hematologic and biochemical parameters.

The purpose of this study was to develop CBC and serum biochemistry reference intervals for the Dunkin Hartley strain and determine age- and sex-related differences. To accomplish this, we accumulated historical CBC and serum biochemistry data from healthy control animals used in our laboratory’s previous studies that represent both males and females of a large age range.

## Materials & methods

### Animals

Retrospective CBC and serum biochemistry data from a total of 145 male (age, 2–15 mo) and 68 female (age, 2–12 mo) Dunkin Hartley guinea pigs were included in this study. At the time of blood collection, male guinea pigs weighed (mean ± SD) 939.29 ± 214.06 g, and female guinea pigs weighed 839.46 ± 200.45 g. All guinea pigs were purchased from Charles River Laboratories (Wilmington, MA) and housed in Fort Collins, Colorado. All animals were singly-housed in 30.80 cm × 59.37 cm × 22.86 cm isolator cages (Maxi-Miser Interchangeable IVC Caging, Thoren, Hazleton, PA) with 0.125-in corn cob bedding (Harlan, Madison, WI) and a red hut (BioServe, French Town, NJ). Caging was changed 2–3 times weekly. Teklad Global Guinea Pig Diet 2040 (Envigo, Madison, WI) and filter-sterilized water were provided *ad libitum*. Hay cubes (PMI Nutrition International LLC, Brentwood, MO) were provided daily. Animal rooms were maintained at a 12:12 h light:dark cycle, 20–26° C temperature, and 30–70% humidity. As per the vendor, all animals were free of Sendai virus, lymphocytic choriomeningitis virus, pneumonia virus of mice, guinea pig adenovirus, guinea pig reovirus, *Helicobacter* spp., *Mycoplasma pulmonis*, and ectoparasites. All original experiments were performed in accordance with *The Guide for the Care and Use of Laboratory Animals* and approved by the Colorado State University Institutional Animal Care and Use Committee.

### Blood collection and analysis

Blood was collected from anesthetized guinea pigs (isoflurane 3–5% in oxygen) from either the cranial vena cava with a 25-gauge needle and 1-mL syringe (n = 40 males; 8 females) or at study harvest via direct cardiac puncture with a 20-gauge butterfly catheter (95 males; 60 females). In a small subset of adult male animals, blood was also collected from an implanted jugular vein catheter while awake (n = 10). Collected blood was placed into 0.5 mL ethylenediaminetetraacetic acid (EDTA) microtubes and red top serum collection tubes. After allowing samples to clot for 20–30 minutes at room temperature, red top tubes were placed into a centrifuge at 3000 x *g* for 15 minutes at 4°C for serum collection. EDTA microtubes and serum aliquots were maintained at 4°C and submitted to the Colorado State University Clinical Pathology Laboratory within 4 hours of collection. CBCs were performed using the Advia 120 hematology analyzer (Siemens, Munich, Germany). Automated parameters included: hemoglobin (Hgb) as measured spectrophotometrically, Hgb (cell) as measured optically, hematocrit (Hct), red blood cell count (RBC), red blood cell distribution width (RDW), mean corpuscular volume (MCV), mean corpuscular hemoglobin concentration (MCHC), cell hemoglobin concentration mean (CHCM), platelets, mean platelet volume (MPV), and white blood cell count (WBC). Manual blood film differentials were performed by trained laboratory staff with experience in guinea pig hematology and identified heterophil, lymphocyte, Foa-Kurloff, monocyte, eosinophil, and basophil percentages and absolute counts. The Roche Cobas 6000 (Basel, Switzerland) was used to measure the following parameters in serum: glucose, blood urea nitrogen (BUN), creatinine, phosphorus, calcium, magnesium, total protein, albumin, globulin, albumin/globulin ratio (A/G), cholesterol, creatine kinase (CK), total bilirubin, alkaline phosphatase (ALP), alanine aminotransferase (ALT), aspartate aminotransferase (AST), gamma glutamyl transferase (GGT), sodium, potassium, chloride, bicarbonate, anion gap, and iron. All analyzers were operating within laboratory established quality assurance protocols that incorporated ASVCP established total allowable error estimates [13,14].

### Statistical analyses

Animals were partitioned into groups based on age. Guinea pigs less than 5 months old were classified as juveniles, and guinea pigs 5 months of age or older were classified as adults. Adult animals were further partitioned into subgroups by sex. Due to low numbers of juvenile females, juveniles were unable to be partitioned by sex.

Following guidelines provided by the ASVCP [3], descriptive statistics (sample size, mean, SD, median, and minimum and maximum values) and reference intervals with confidence intervals were determined for each group using Reference Value Advisor v2.1 [15]. Histograms were evaluated to assess distribution and outliers. Outliers identified by the Tukey test were removed. After outlier removal, normality was assessed using the Anderson-Darling test with P value < 0.05 considered statistically significant. For parameters with ≥ 40 reference samples, the nonparametric method was performed to determine the 2.5^th^ and 97.5^th^ percentile of each parameter to serve as the lower and upper limits of the reference interval, respectively. The 90% confidence intervals of the lower and upper limits of the reference interval were then determined using the bootstrap method. For parameters with < 40 reference samples, reference intervals with 90% confidence intervals of the reference limits were calculated by parametric or robust methods. If the distribution was non-Gaussian, the data were transformed using the Box-Cox method and rechecked for distribution.

Data were analyzed using Prism (version 8.4.0, GraphPad Software, La Jolla, CA). Normality was assessed using the D’Agostino-Pearson normality test. An unpaired t-test for normally distributed data or a nonparametric Mann-Whitney test for non-normally distributed data was used to determine age- and sex-associated differences. Age correlation was determined using the Spearman coefficient. Results were considered statistically significant with a P value < 0.05.

## Results

### Hematology

Descriptive statistics and reference intervals were established for hematology parameters of 68 juvenile (52 males; 16 females) and 144 adult (93 males; 51 females) Dunkin Hartley guinea pigs (Table 1). Manual WBC differential counts were unavailable from 12 adult females. Thus, reference intervals for heterophil, lymphocyte, monocyte, eosinophil, and basophil percentages and counts were calculated from 132 adults. Compared to juveniles, adults had significantly higher Hct (median difference 1%; 95% CI 0–2; P = 0.0127), RDW (median difference 0.4%; 95% CI 0–0.7; P = 0.0482), MPV (median difference 0.7 fl; 95% CI 0.3–0.7; P < 0.0001), WBC (median difference 0.8 × 10^3^/μL; 95% CI 0.3–1.1; P = 0.0018), heterophil % (mean difference 8%; 95% CI 5–11; P < 0.0001), heterophils (median difference 0.732 × 10^3^/μL; 95% CI 0.359–0.896; P < 0.0001), monocytes (median difference 0.046 × 10^3^/μL; 95% CI 0.006–0.090; P = 0.0227), eosinophil % (median difference 1%; 95% CI 0–1; P = 0.0106), and eosinophils (median difference 0.042 × 10^3^/μL; 95% CI 0.007–0.058; P = 0.0048). Juveniles had higher MCHC (mean difference 1 g/dL; 95% CI 0–1; P = 0.0008), CHCM (mean difference 1 g/dL; 95% CI 0–1; P = 0.003), platelets (median difference 32 × 10^3^/μL; 95% CI 15–80; P = 0.0039), lymphocyte % (mean difference 11%; 95% CI 7–14; P < 0.0001), and lymphocytes (median 0.103 × 10^3^/μL; 95% CI 0.016–0.462; P = 0.0357) compared to adults.

**Table 1:** Summary data and reference intervals for hematology parameters of juvenile (< 5 mo) and adult (≥ 5 mo) Hartley guinea pigs.

Analyte	Age	N	Mean	SD	Median	Min	Max	P-value	Distribution	Method	RI	90% CI of LRL	90% CI of URL	P-value (Juveniles vs. Adults)
**Hgb (g/dL)**	Juveniles	67	15.2	0.7	15.1	13.4	16.7	0.371	G	NP	13.8–16.6	13.4–14.2	16.3–16.7	0.2668
	Adults	144	15.3	0.9	15.4	11.9	17.3	0.001	NG	NP	13.4–16.7	11.9–13.6	16.5–17.3	
**Hgb (cell) (g/dL)**	Juveniles	67	15.1	0.8	15.2	13.5	16.9	0.496	G	NP	13.6–16.6	13.5–13.9	16.2–16.9	0.2878
	Adults	144	15.2	1.0	15.4	11.9	17.4	0.002	NG	NP	12.9–17.0	11.9–13.4	16.6–17.4	
**Hct (%)**	Juveniles	67	46	2	46	41	52	0.012	NG	NP	41–52	41–42	50–52	0.0127^a^
	Adults	144	47	3	47	38	53	0.000	NG	NP	41–51	38–42	51–53	
**RBC (**× **10**^**6**^**/μL)**	Juveniles	68	5.59	0.32	5.56	4.76	6.53	0.599	G	NP	4.93–6.30	4.76–5.12	6.12–6.53	0.2247
	Adults	144	5.62	0.37	5.66	4.16	6.42	0.028	NG	NP	4.79–6.20	4.16–4.98	6.15–6.42	
**MCV (fl)**	Juveniles	68	83	3	83	77	90	0.062	G	NP	78–89	77–78	87–90	0.2222
	Adults	143	84	3	84	78	94	0.000	NG	NP	79–89	78–80	88–94	
**RDW (%)**	Juveniles	67	13.0	1.6	12.5	11.0	19.4	0.000	NG	NP	11.1–17.2	11.0–11.2	15.4–19.4	0.0482^a^
	Adults	143	13.0	0.9	12.9	11.1	16.0	0.000	NG	NP	11.5–15.3	11.1–11.7	15.0–16.0	
**MCHC (g/dL)**	Juveniles	68	33	1	33	31	35	0.000	NG	NP	31–35	31–32	34–35	0.0008^c^
	Adults	144	32	1	32	30	35	0.000	NG	NP	31–34	30–31	34–35	
**CHCM (g/dL)**	Juveniles	68	33	1	33	30	35	0.000	NG	NP	31–35	30–31	34–35	0.0030^b^
	Adults	143	32	1	32	30	35	0.000	NG	NP	31–34	30–31	34–35	
**Platelets (**× **10**^**3**^**/μL)**	Juveniles	68	539	113	519	297	856	0.055	G	NP	332–801	297–363	735–856	0.0039^b^
	Adults	143	489	124	487	112	943	0.002	NG	NP	259–800	112–298	703–943	
**MPV (fl)**	Juveniles	68	8.0	0.7	7.9	6.6	9.2	0.040	NG	NP	6.8–9.1	6.6–7.0	9.0–9.2	< 0.0001^d^
	Adults	144	8.4	0.5	8.6	7.0	9.3	0.000	NG	NP	7.3–9.2	7.0–7.5	9.1–9.3	
**WBC (**× **10**^**3**^**/μL)**	Juveniles	68	5.1	1.5	4.9	2.2	9.6	0.000	NG	NP	2.6–9.4	2.2–3.2	8.7–9.6	0.0018^b^
	Adults	144	5.7	1.8	5.7	1.9	11.7	0.008	NG	NP	2.7–10.3	1.9–3.1	8.9–11.7	
**Heterophils (%)**	Juveniles	68	41	10	40	18	64	0.343	G	NP	18–61	18–26	56–64	< 0.0001^d^
	Adults	132	49	11	49	23	79	0.380	G	NP	29–72	23–33	66–79	
**Heterophils (**× **10**^**3**^**/μL)**	Juveniles	68	2.134	1.002	1.888	0.396	5.248	0.000	NG	NP	0.738–5.219	0.396–1.012	4.349–5.248	< 0.0001^d^
	Adults	130	2.750	1.078	2.620	0.437	6.804	0.001	NG	NP	1.054–5.403	0.437–1.333	4.902–6.804	
**Lymphocytes (%)**	Juveniles	68	52	9	51	31	71	0.761	G	NP	33–71	31–38	68–71	< 0.0001^d^
	Adults	132	41	12	41	14	70	0.243	G	NP	18–63	14–26	59–70	
**Lymphocytes (**× **10**^**3**^**/μL)**	Juveniles	68	2.553	0.702	2.339	1.414	4.644	0.000	NG	NP	1.521–4.387	1.414–1.702	3.866–4.644	0.0357^a^
	Adults	132	2.308	0.827	2.236	0.476	4.816	0.087	G	NP	0.775–4.177	0.476–1.140	3.577–4.816	
**Foa-Kurloff Cells (%)**	Juveniles	67	3	3	2	0	9	0.000	NG	NP	0–9	0–0	8–9	0.1032
	Adults	132	3	2	3	0	11	0.000	NG	NP	0–9	0–0	8–11	
**Foa-Kurloff Cells (**× **10**^**3**^**/μL)**	Juveniles	68	0.150	0.151	0.126	0	0.576	0.000	NG	NP	0–0.512	0–0	0.415–0.576	0.0504
	Adults	132	0.192	0.163	0.156	0	0.757	0.000	NG	NP	0–0.710	0–0	0.500–0.757	
**Monocytes (%)**	Juveniles	68	4	2	3	0	9	0.000	NG	NP	0–9	0–1	8–9	0.3164
	Adults	131	4	3	4	0	14	0.000	NG	NP	0–10	0–1	8–14	
**Monocytes (**× **10**^**3**^**/μL)**	Juveniles	67	0.172	0.124	0.159	0	0.558	0.000	NG	NP	0–0.503	0–0.033	0.415–0.558	0.0227^a^
	Adults	131	0.239	0.195	0.205	0	1.020	0.000	NG	NP	0–0.792	0–0.019	0.696–1.020	
**Eosinophils (%)**	Juveniles	67	2	1	1	0	5	0.000	NG	NP	0–5	0–0	4–5	0.0106^a^
	Adults	130	2	2	2	0	8	0.000	NG	NP	0–7	0–0	6–8	
**Eosinophils (**× **10**^**3**^**/μL)**	Juveniles	67	0.074	0.067	0.060	0	0.265	0.000	NG	NP	0–0.253	0–0	0.210–0.265	0.0048^b^
	Adults	128	0.117	0.101	0.102	0	0.442	0.000	NG	NP	0–0.363	0–0	0.330–0.442	
**Basophils (%)**	Juveniles	68	0	1	0	0	2	0.000	NG	NP	0–2	0–0	1–2	0.3021
	Adults	130	0	1	0	0	2	0.000	NG	NP	0–2	0–0	2–2	
**Basophils (**× **10**^**3**^**/μL)**	Juveniles	68	0.012	0.027	0	0	0.112	0.000	NG	NP	0–0.102	0–0	0.069–0.112	0.3041
	Adults	128	0.016	0.030	0	0	0.108	0.000	NG	NP	0–0.102	0–0	0.089–0.108	

^a^*P* < 0.05

^b^*P* < 0.01

^c^*P* < 0.001

^d^*P* < 0.0001 between values for juvenile and adult Hartley guinea pigs. Abbreviations: G, Gaussian; NG, non-Gaussian; NP, nonparametric; RI, reference interval.

Age correlation of hematology parameters in male and female guinea pigs is shown in Table 2. In both sexes, MPV (males: r = 0.4555; females: r = 0.5114), heterophil % (males: r = 0.3237; females: r = 0.5893) and heterophils (males: r = 0.2996; females: r = 0.5587) were positively correlated with age, and CHCM (males: r = -0.2777; females: r = -0.3660) and lymphocyte % (males: r = -0.4704; females: r = -0.5799) were negatively correlated with age. In males alone, Hct (r = 0.1942), RDW (r = 0.2108), WBC (r = 0.1802), Foa-Kurloff cell % (r = 0.3274), monocytes (r = 0.1937), eosinophils % (r = 0.1972), and eosinophils (r = 0.2401) were positively correlated with age, while MCHC (r = -0.2498), platelets (r = -0.2647), and lymphocytes (r = -0.2186) were negatively correlated. MCV (r = 0.4211) was positively correlated with age in females.

**Table 2:** Spearman correlation (r) of hematology parameters with age in 145 male and 67 female Dunkin Hartley guinea pigs.

Analyte	Males	Females
Hgb (g/dL)	0.09060	0.0848
Hgb (cell) (g/dL)	0.03184	0.0255
Hct (%)	0.1942^a^	0.1354
RBC (× 10^6^/μL)	0.1360	-0.0576
MCV (fl)	0.05356	0.4211^c^
RDW (%)	0.2108^a^	-0.1209
MCHC (g/dL)	-0.2498^b^	-0.2016
CHCM (g/dL	-0.2777^c^	-0.3660^b^
Platelets (× 10^3^/μL)	-0.2647^b^	-0.1259
MPV (fl)	0.4555^d^	0.5114^d^
WBC (× 10^3^/μL)	0.1802^a^	0.1620
Heterophils (%)	0.3237^d^	0.5893^d^
Heterophils (× 10^3^/μL)	0.2996^c^	0.5587^d^
Lymphocytes (%)	-0.4704^d^	-0.5799^d^
Lymphocytes (× 10^3^/μL)	-0.2186^b^	-0.2508
Foa-Kurloff Cells (%)	0.3274^d^	-0.2113
Foa-Kurloff Cells (× 10^3^/μL)	0.3193^d^	-0.1643
Monocytes (%)	0.1311	0.1191
Monocytes (× 10^3^/μL)	0.1937^a^	0.2224
Eosinophils (%)	0.1972^a^	0.2249
Eosinophils (× 10^3^/μL)	0.2401^b^	0.2165
Basophils (%)	0.07390	0.01767
Basophils (× 10^3^/μL)	0.06626	0.0280

^a^*P* < 0.05

^b^*P* < 0.01

^c^*P* < 0.001

^d^*P* < 0.0001.

Hematology reference intervals for adult guinea pigs were partitioned by sex (Table 3). Since manual WBC differential counts were unavailable from 12 adult females, reference intervals for heterophil, lymphocyte, monocyte, eosinophil, and basophil percentages and counts were calculated from 39 females. Males had significantly higher Hgb (median difference 0.5 g/dL; 95% CI 0.3–0.9; P = 0.003), Hgb (cell) (mean difference 0.5 g/dL; 95% CI 0.1–0.8; P = 0.0033), Hct (median difference 2%; 95% CI 0–2; P = 0.0231), RBC (median difference 0.31 × 10^6^/μL; 95% CI 0.21–0.43; P < 0.0001), MCHC (median difference 1 g/dL; 95% CI 0–1; P = 0.0002), WBC (median difference 0.4 × 10^3^/μL; 0.1–1.2; P = 0.0179), heterophil % (median difference 11%; 95% CI 5–13; P < 0.0001), heterophils (median difference 0.794 × 10^3^/μL; 95% CI 0.405–1.054; P = 0.0001), Foa-Kurloff cell % (mean difference 2%; 95% CI 0–2; P = 0.0084), and Foa-Kurloff cells (median difference 0.075 × 10^3^/μL; 95% CI 0.010–0.120; P = 0.0095) compared to females. Females had higher MCV (median difference 3 fl; 95% CI 2–4; P < 0.0001), RDW (median difference 0.4%; 95% CI 0.3–0.8; P = 0.0002), platelets (median difference 77 × 10^3^/μL; 95% CI 45–126; P < 0.0001), MPV (median difference 0.5 fl; 95% CI 0.3–0.6; P < 0.0001), lymphocyte % (median difference 8%; 95% CI 2–12; P = 0.0017), eosinophil % (median difference 2%; 95% CI 1–3; P < 0.0001), and eosinophils (median difference 0.071 × 10^3^/μL; 95% CI 0.036–0.121; P = 0.0001) than males.

**Table 3:** Summary data and reference intervals for hematology parameters of adult male and female Hartley guinea pigs.

Analyte	Sex	N	Mean	SD	Median	Min	Max	P-value	Distribution	Method	RI	90% CI of LRL	90% CI of URL	P-value (Males vs. Females)
**Hgb (g/dL)**	Males	93	15.5	0.9	15.6	12.4	17.3	0.022	NG	NP	13.4–16.9	12.4–13.8	16.6–17.3	0.0003^c^
	Females	50	14.9	0.9	15.1	13.3	16.7	0.063	G	NP	13.3–16.6	13.3–13.5	16.1–16.7	
**Hgb (cell) (g/dL)**	Males	93	15.4	0.9	15.5	12.6	17.4	0.106	G	NP	13.2–17.3	12.6–13.9	16.8–17.4	0.0033^b^
	Females	51	14.9	1.0	15.0	11.9	16.6	0.015	NG	NP	12.1–16.5	11.9–13.2	16.1–16.6	
**Hct (%)**	Males	93	47	3	48	38	53	0.000	NG	NP	41–52	38–43	51–53	0.0231^a^
	Females	51	46	3	46	40	52	0.195	G	NP	40–51	40–41	50–52	
**RBC (**× **10**^**6**^**/μL)**	Males	93	5.73	0.33	5.76	4.59	6.42	0.042	NG	NP	4.83–6.24	4.59–5.17	6.17–6.42	< 0.0001^d^
	Females	51	5.41	0.36	5.45	4.16	6.18	0.259	G	NP	4.35–6.11	4.16–4.92	5.88–6.18	
**MCV (fl)**	Males	93	83	2	83	78	89	0.000	NG	NP	78–88	78–80	86–89	< 0.0001^d^
	Females	51	86	3	86	79	95	0.028	NG	NP	79–95	79–81	91–95	
**RDW (%)**	Males	93	12.8	0.8	12.8	11.1	15.3	0.001	NG	NP	11.4–15.0	11.1–11.6	14.2–15.3	0.0002^c^
	Females	51	13.5	1.1	13.2	11.8	17.2	0.020	NG	NP	11.8–16.8	11.8–12.0	15.4–17.2	
**MCHC (g/dL)**	Males	92	33	1	33	32	35	0.000	NG	NP	32–34	32–32	34–35	0.0002^c^
	Females	51	32	1	32	30	34	0.000	NG	NP	30–34	30–31	34–34	
**CHCM (g/dL)**	Males	92	32	1	32	31	35	0.000	NG	NP	31–34	31–31	33–35	0.1094
	Females	51	32	1	32	30	34	0.000	NG	NP	30–34	30–30	34–34	
**Platelets (**× **10**^**3**^**/μL)**	Males	93	454	99	470	112	688	0.108	G	NP	197–636	112–288	604–688	< 0.0001^d^
	Females	51	562	155	547	333	1025	0.012	NG	NP	335–1000	333–352	837–1025	
**MPV (fl)**	Males	93	8.3	0.5	8.3	7.0	9.2	0.014	NG	NP	7.1–9.1	7.0–7.3	9.0–9.2	< 0.0001^d^
	Females	50	8.8	0.3	8.8	7.7	9.3	0.010	NG	NP	7.7–9.3	7.7–8.3	9.3–9.3	
**WBC (**× **10**^**3**^**/μL)**	Males	93	6.0	1.8	5.8	2.0	11.7	0.041	NG	NP	3.0–10.7	2.0–3.2	9.5–11.7	0.0179^a^
	Females	51	5.3	1.5	5.4	1.9	9.1	0.388	G	NP	2.1–9.0	1.9–3.0	7.3–9.1	
**Heterophils (%)**	Males	93	51	10	51	31	73	0.809	G	NP	33–72	31–35	68–73	0.0001^c^
	Females	39	43	12	40	23	79	0.027	NG	TS	25–75	23–28	65–88	
**Heterophils (**× **10**^**3**^**/μL)**	Males	91	2.972	1.085	2.907	1.156	6.804	0.006	NG	NP	1.324–5.463	1.156–1.426	5.157–6.804	0.0001^c^
	Females	38	2.161	0.765	2.113	0.437	4.277	0.101	G	US	0.590–3.731	0.248–0.955	3.369–4.101	
**Lymphocytes (%)**	Males	93	39	10	40	14	63	0.755	G	NP	18–59	14–24	56–63	0.0017^b^
	Females	39	46	13	48	14	70	0.099	G	US	18–73	12–25	67–79	
**Lymphocytes (**× **10**^**3**^**/μL)**	Males	93	2.283	0.769	2.223	0.560	4.816	0.153	G	NP	0.806–3.695	0.560–1.168	3.540–4.816	0.5240
	Females	39	2.367	0.959	2.420	0.476	4.450	0.519	G	US	0.402–4.333	0–0.865	3.885–4.782	
**Foa-Kurloff Cells (%)**	Males	93	4	3	4	0	11	0.008	NG	NP	0–9	0–0	9–11	0.0084^b^
	Females	39	2	2	2	0	6	0.025	NG	TS	0–4	0–1	4–5	
**Foa-Kurloff Cells (**× **10**^**3**^**/μL)**	Males	93	0.215	0.174	0.183	0	0.757	0.000	NG	NP	0–0.713	0–0	0.513–0.757	0.0095^b^
	Females	39	0.137	0.119	0.108	0	0.396	0.003	NG	TS	0–0.436	0–0	0.349–0.521	
**Monocytes (%)**	Males	93	4	3	4	0	11	0.001	NG	NP	0–10	0–1	9–11	0.1418
	Females	37	3	2	3	0	7	0.056	NG	US	1–7	0–0	6–8	
**Monocytes (**× **10**^**3**^**/μL)**	Males	93	0.259	0.210	0.210	0	1.020	0.000	NG	NP	0.004–0.930	0–0.034	0.710–1.020	0.2136
	Females	38	0.192	0.144	0.163	0	0.574	0.074	G	NP	0–0.487	0–0	0.409–0.553	
**Eosinophils (%)**	Males	93	2	1	2	0	7	0.000	NG	NP	0–6	0–0	4–7	< 0.0001^d^
	Females	38	4	3	4	0	11	0.112	G	UR	0–9	0–0	8–10	
**Eosinophils (**× **10**^**3**^**/μL)**	Males	93	0.097	0.089	0.077	0	0.442	0.000	NG	NP	0–0.339	0–0	0.278–0.442	0.0001^c^
	Females	37	0.195	0.157	0.148	0	0.712	0.003	NG	TS	0–0.618	0–0.019	0.480–0.783	
**Basophils (%)**	Males	93	0	1	0	0	2	0.000	NG	NP	0–2	0–0	1–2	0.8752
	Females	37	0	1	0	0	2	0.000	NG	US	0–2	0–0	1–2	
**Basophils (**× **10**^**3**^**/μL)**	Males	91	0.015	0.027	0	0	0.103	0.000	NG	NP	0–0.098	0–0	0.080–0.103	0.9139
	Females	37	0.019	0.037	0	0	0.128	0.000	NG	US	0–0.096	0–0	0.060–0.119	

^a^*P* < 0.05

^b^*P* < 0.01

^c^*P* < 0.001

^d^*P* < 0.0001 between values for adult male and female Hartley guinea pigs. Abbreviations: G, Gaussian; NG, non-Gaussian; NP, nonparametric; US, untransformed standard; UR, untransformed robust; TS, transformed standard; RI, reference interval.

### Serum biochemistry

Descriptive statistics and reference intervals were established for serum biochemistry analytes of 49 juveniles (41 males; 8 females) and 145 adult (93 males; 52 females) Dunkin Hartley guinea pigs (Table 4). Magnesium was unavailable from 11 adult males. Iron was unavailable from 28 juvenile males and 23 adult males. Additionally, there was insufficient blood volume to complete testing of all analytes in some animals: glucose (2 juveniles; 1 adult), BUN (2 juveniles; 1 adult), creatinine (1 juvenile; 1 adult), phosphorus (2 juveniles), calcium (3 juveniles), magnesium (3 juveniles), total protein (2 juveniles), globulin (2 juveniles), A/G (2 juveniles), cholesterol (1 juvenile; 1 adult), total bilirubin (2 juveniles), ALP (1 juvenile), ALT (1 juvenile), GGT (1 juvenile), bicarbonate (2 juveniles), anion gap (2 juveniles; 1 adult), and calculated osmolality (2 juveniles; 1 adult).

**Table 4:** Summary data and reference intervals for serum biochemistry parameters of juvenile (< 5 mo) and adult (≥ 5 mo) Hartley guinea pigs.

Analyte	Age	N	Mean	SD	Median	Min	Max	P-value	Distribution	Method	RI	90% CI of LRL	90% CI of URL	P-value (Juveniles vs. Adults)
**Glucose (mg/dL)**	Juveniles	47	203	47	201	125	415	0.020	NG	NP	127–384	125–153	256–415	< 0.0001^d^
	Adults	137	235	60	227	123	454	0.000	NG	NP	128–427	123–146	374–454	
**BUN (mg/dL)**	Juveniles	47	18	2	18	11	23	0.048	NG	NP	11–23	11–14	21–23	< 0.0001^d^
	Adults	143	21	3	21	13	33	0.010	NG	NP	16–29	13–16	27–33	
**Creatinine (mg/dL)**	Juveniles	47	0.3	0.1	0.3	0.2	0.4	0.000	NG	NP	0.2–0.4	0.2–0.2	0.3–0.4	< 0.0001^d^
	Adults	144	0.4	0.1	0.3	0.2	0.6	0.000	NG	NP	0.2–0.5	0.2–0.3	0.5–0.6	
**Phosphorus (mg/dL)**	Juveniles	47	6.1	1.1	6.1	3.5	8.6	0.165	G	NP	3.6–8.5	3.5–4.5	7.9–8.6	< 0.0001^d^
	Adults	145	5.1	0.8	5.0	3.4	7.6	0.019	NG	NP	3.7–6.9	3.4–3.8	6.4–7.6	
**Calcium (mg/dL)**	Juveniles	46	10.7	0.6	10.7	8.9	11.8	0.285	G	NP	9.1–11.8	8.9–9.9	11.6–11.8	< 0.0001^d^
	Adults	145	11.3	0.5	11.3	10.0	12.9	0.007	NG	NP	10.5–12.5	10.0–10.6	12.2–12.9	
**Magnesium (mg/dL)**	Juveniles	46	3.3	0.5	3.2	2.5	4.6	0.021	NG	NP	2.5–4.6	2.5–2.6	4.3–4.6	< 0.0001^d^
	Adults	134	3.6	0.5	3.6	2.2	5.1	0.174	G	NP	2.7–4.6	2.2–2.9	4.4–5.1	
**Total Protein (g/dL)**	Juveniles	46	4.8	0.3	4.8	4.0	5.3	0.150	G	NP	4.0–5.3	4.0–4.3	5.2–5.3	< 0.0001^d^
	Adults	145	5.4	0.3	5.3	4.7	6.4	0.030	NG	NP	4.7–6.1	4.7–4.8	5.9–6.4	
**Albumin (g/dL)**	Juveniles	47	3.0	0.1	3.0	2.7	3.3	0.000	NG	NP	2.7–3.3	2.7–2.8	3.2–3.3	< 0.0001^d^
	Adults	145	3.2	0.2	3.2	2.7	3.7	0.000	NG	NP	2.8–3.6	2.7–2.8	3.5–3.7	
**Globulin (g/dL)**	Juveniles	47	1.8	0.2	1.8	1.4	2.4	0.205	G	NP	1.4–3.4	1.4–1.4	2.1–2.4	< 0.0001^d^
	Adults	145	2.2	0.2	2.2	1.7	2.9	0.000	NG	NP	1.8–2.6	1.7–1.9	2.5–2.9	
**A/G Ratio**	Juveniles	47	1.66	0.19	1.65	1.40	2.10	0.015	NG	NP	1.40–2.10	1.40–1.40	1.90–2.10	< 0.0001^d^
	Adults	145	1.45	0.13	1.45	1.04	1.80	0.028	NG	NP	1.20–1.75	1.04–1.27	1.70–1.80	
**Cholesterol (mg/dL)**	Juveniles	48	31	7	31	19	56	0.022	NG	NP	20–54	19–23	44–56	0.0100^b^
	Adults	140	37	14	35	12	86	0.000	NG	NP	16–79	12–19	68–86	
**CK (IU/L)**	Juveniles	47	434	278	380	141	1211	0.000	NG	NP	142–1200	141–161	1083–1211	0.1648
	Adults	139	560	437	425	107	2269	0.000	NG	NP	121–1897	107–141	1505–2269	
**Total Bilirubin (mg/dL)**	Juveniles	47	0	0	0	0	0	-	-	NP	0–0	0–0	0–0	> 0.9999
	Adults	144	0	0	0	0	0	-	-	NP	0–0	0–0	0–0	
**ALP (IU/L)**	Juveniles	47	188	64	181	89	322	0.057	G	NP	89–320	89–97	284–322	< 0.0001^d^
	Adults	145	79	36	72	29	205	0.000	NG	NP	34–167	29–37	159–205	
**ALT (IU/L)**	Juveniles	48	35	10	33	12	69	0.002	NG	NP	15–66	12–25	52–69	0.0827
	Adults	142	38	11	36	15	77	0.000	NG	NP	18–70	15–23	59–77	
**AST (IU/L)**	Juveniles	48	44	20	39	22	108	0.000	NG	NP	23–108	22–27	84–108	< 0.0001^d^
	Adults	141	67	36	54	22	203	0.000	NG	NP	25–171	22–29	134–203	
**GGT (IU/L)**	Juveniles	48	11	6	10	0	27	0.003	NG	NP	1–27	0–5	23–27	< 0.0001^d^
	Adults	145	18	7	17	0	37	0.004	NG	NP	7–34	0–8	31–37	
**Sodium (mEQ/L)**	Juveniles	49	136	2	136	130	140	0.000	NG	NP	130–140	130–131	139–140	0.8469
	Adults	145	136	3	137	127	144	0.000	NG	NP	129–141	127–131	140–144	
**Potassium (mEQ/L)**	Juveniles	49	5.30	1.06	5.15	3.85	9.00	0.001	NG	NP	3.89–8.88	3.85–4.16	7.24–9.00	0.1433
	Adults	143	5.06	0.88	4.88	3.66	7.41	0.000	NG	NP	3.90–7.04	3.66–3.99	6.90–4.41	
**Chloride (mEQ/L)**	Juveniles	49	101.3	2.5	101.4	94.7	106.7	0.885	G	NP	95.0–106.5	94.7–98.1	105.0–106.7	0.3990
	Adults	145	100.7	3.3	101.2	88.5	107.3	0.002	NG	NP	91.7–107.1	88.5–95.2	105.2–107.3	
**Bicarbonate (mEQ/L)**	Juveniles	46	21.2	3.4	21.2	14.8	28.6	0.578	G	NP	15.0–28.5	14.8–16.2	26.2–28.6	< 0.0001^d^
	Adults	144	24.4	2.5	24.6	18.5	31.3	0.677	G	NP	19.4–29.4	18.5–20.1	28.4–31.3	
**Anion Gap (mmol/L)**	Juveniles	47	19	3	18	11	26	0.273	G	NP	12–26	11–14	24–26	< 0.0001^d^
	Adults	142	16	2	16	12	22	0.000	NG	NP	13–21	12–13	21–22	
**Calculated Osmolality (mOsm/kg)**	Juveniles	47	281	5	281	268	289	0.090	G	NP	269–289	268–271	288–289	0.0001^c^
	Adults	144	284	5	284	269	302	0.097	G	NP	273–294	269–275	292–302	
**Iron (μg/dL)**	Juveniles	21	293	29	288	232	358	0.331	G	US	231–356	211–252	338–377	0.5212
	Adults	122	299	41	298	186	405	0.614	G	NP	215–391	186–238	367–405	

^a^*P* < 0.05

^b^*P* < 0.01

^c^*P* < 0.001

^d^*P* < 0.0001 between values for juvenile and adult Hartley guinea pigs. Abbreviations: G, Gaussian; NG, non-Gaussian; NP, nonparametric; US, untransformed standard; RI, reference interval.

Significant differences in biochemistry parameters between juveniles and adults are shown in Table 4. Glucose (median difference 26 mg/dL; 95% CI 16–44; P < 0.0001), BUN (median difference 3 mg/dL; 95% CI 2–4; P < 0.0001), creatinine (median difference 0.0 mg/dL; 95% CI 0.1–0.1; P < 0.0001), calcium (median difference 0.6 mg/dL; 95% CI 0.4–0.7; P < 0.0001), magnesium (median difference 0.4 mg/dL; 95% CI 0.2–0.5; P < 0.0001), total protein (mean difference 0.6 g/dL; 95% CI 0.5–0.7; P < 0.0001), albumin (mean difference 0.2 g/dL; 95% CI 0.1–0.3; P < 0.0001), globulin (mean difference 0.4 g/dL; 95% CI 0.3–0.5; P < 0.0001), cholesterol (median difference 4 mg/dL; 95% CI 1–7; P = 0.01), AST (median difference 15 IU/L; 95% CI 9–23; P < 0.0001), GGT (median difference 7 IU/L; 95% CI 4–9; P < 0.0001), bicarbonate (mean difference 3.2 mEQ/L; 95% CI 2.3–4.2; P < 0.0001), and calculated osmolality (mean difference 3 mOsm/kg; 95% CI 2–5; P = 0.0001) were significantly increased in adults compared to juveniles. Phosphorus (median difference 1.1 mg/dL; 95% CI 0.8–1.3; P < 0.0001), A/G (mean difference 0.21; 95% CI 0.16–0.26; P < 0.0001), ALP (median difference 109 IU/L; 95% CI 85–127; P < 0.0001), and anion gap (median difference 2 mmol/L; 95% CI 2–4; P < 0.0001) were significantly increased in juveniles compared to adults.

Age correlation of serum biochemistry parameters in males and females is presented in Table 5. In both males and females, BUN (males: r = 0.3729; females: r = 0.4789), creatinine (males: r = 0.6504; females: r = 0.2947), calcium (males: r = 0.4971; females: r = 0.3740), total protein (males: r = 0.6481; females: r = 0.5333), albumin (males: r = 0.4198; females: r = 0.3804), globulin (males: r = 0.6557; females: r = 0.5455), and AST (males: r = 0.3374; females: r = 0.3494) were positively correlated with age, and phosphorus (males: r = -0.5403; females: r = -0.2627), A/G (males: r = -0.5709; females: r = -0.3273), and ALP (males: r = -0.7074; females: r = -0.7501) were negatively correlated with age. Glucose (r = 0.4273), magnesium (r = 0.3933), cholesterol (r = 0.1776), CK (r = 0.2006), ALT (r = 0.2023), GGT (r = 0.3974), and bicarbonate (r = 0.4356) increased with age, whereas potassium (r = -0.1780) and anion gap (r = -0.4931) decreased with age in males. In females, sodium (r = 0.2650) and iron (r = 0.6484) increased with age.

**Table 5:** Spearman correlation (r) of serum biochemistry parameters with age in male and female Dunkin Hartley guinea pigs.

Analyte	Males	Females
Glucose (mg/dL)	0.4273^d^	0.0817
BUN (mg/dL)	0.3729^d^	0.4789^c^
Creatinine (mg/dL)	0.6504^d^	0.2947^a^
Phosphorus (mg/dL)	-0.5403^d^	-0.2627^a^
Calcium (mg/dL)	0.4971^d^	0.3740^b^
Magnesium (mg/dL)	0.3933^d^	0.0363
Total Protein (g/dL)	0.6481^d^	0.5333^d^
Albumin (g/dL)	0.4198^d^	0.3804^b^
Globulin (g/dL)	0.6557^d^	0.5455^d^
A/G Ratio	-0.5709^d^	-0.3273^a^
Cholesterol (mg/dL)	0.177^a^	0.0892
CK (IU/L)	0.2006^a^	0.1707
Total Bilirubin (mg/dL)	-	-
ALP (IU/L)	-0.7074^d^	-0.7501^d^
ALT (IU/L)	0.2023^a^	0.04274
AST (IU/L)	0.3374^d^	0.3494^b^
GGT (IU/L)	0.3974^d^	0.2274
Sodium (mEQ/L)	0.1533	0.2650^a^
Potassium (mEQ/L)	-0.1780^a^	0.1865
Chloride (mEQ/L)	0.1291	0.2298
Bicarbonate (mEQ/L)	0.4356^d^	-0.1111
Anion Gap (mmol/L)	-0.4931^d^	0.0681
Calculated Osmolality (mOsm/kg)	0.3648^d^	0.4568^c^
Iron (μg/dL)	0.0623	0.6484^d^

^a^*P* < 0.05

^b^*P* < 0.01

^c^*P* < 0.001

^d^*P* < 0.0001.

Adult reference intervals for serum biochemistry were partitioned by sex (Table 6). Magnesium and iron were unavailable from 11 and 23 males, respectively. Due to low blood sample volumes, the following analytes were not tested in all animals: glucose (n = 1 male), BUN (1 male), creatinine (1 male), cholesterol (1 male), anion gap (1 female), and calculated osmolality (1 female). Outliers removed from males included glucose (n = 2), BUN (1), CK (4), total bilirubin (1), ALT (2), AST (2), potassium (1), and chloride (1). Glucose (n = 2), CK (3), ALT (1), AST (2), and bicarbonate (1) outliers were removed from females.

**Table 6:** Summary data and reference intervals for serum biochemistry parameters of adult male and female Hartley guinea pigs.

Analyte	Sex	N	Mean	SD	Median	Min	Max	P-value	Distribution	Method	RI	90% CI of LRL	90% CI of URL	P-value (Males vs. Females)
**Glucose (mg/dL)**	Males	90	229	55	227	123	411	0.002	NG	NP	126–392	123–140	315–411	0.6527
	Females	50	260	84	232	159	475	0.000	NG	NP	163–473	159–184	454–475	
**BUN (mg/dL)**	Males	91	21	3	21	16	33	0.030	NG	NP	16–29	16–16	26–33	0.9909
	Females	52	21	4	21	13	32	0.348	G	NP	14–31	13–16	27–32	
**Creatinine (mg/dL)**	Males	92	0.4	0.1	0.4	0.2	0.6	0.000	NG	NP	0.2–0.5	0.2–0.3	0.5–0.6	0.0870
	Females	52	0.3	0.1	0.3	0.2	0.5	0.000	NG	NP	0.2–0.5	0.2–0.3	0.4–0.5	
**Phosphorus (mg/dL)**	Males	93	5.0	0.7	5.0	3.6	6.9	0.289	G	NP	3.7–6.4	3.6–3.8	6.2–6.9	0.0838
	Females	52	5.2	1.0	5.1	3.4	7.6	0.237	G	NP	3.5–7.5	3.4–4.0	6.9–7.6	
**Calcium (mg/dL)**	Males	93	11.3	0.4	11.3	10.4	12.5	0.118	G	NP	10.5–12.4	10.4–10.6	12.0–12.5	0.4169
	Females	52	11.3	0.6	11.3	10.0	12.9	0.058	G	NP	10.1–12.8	10.0–10.5	12.4–12.9	
**Magnesium (mg/dL)**	Males	82	3.6	0.5	3.7	2.5	5.1	0.256	G	NP	2.7–5.1	2.5–2.9	4.5–5.1	0.1709
	Females	52	3.5	0.4	3.5	2.2	4.4	0.389	G	NP	2.4–4.3	2.2–2.8	4.1–4.4	
**Total Protein (g/dL)**	Males	93	5.3	0.3	5.3	4.7	5.8	0.018	NG	NP	4.7–5.8	4.7–4.8	5.7–5.8	0.0002^c^
	Females	52	5.5	0.4	5.5	4.7	6.4	0.245	G	NP	4.7–6.3	4.7–5.0	6.1–6.4	
**Albumin (g/dL)**	Males	93	3.1	0.2	3.1	2.7	3.4	0.000	NG	NP	2.8–6.4	2.7–2.8	3.3–3.4	< 0.0001^d^
	Females	52	3.3	0.2	3.3	2.7	3.7	0.112	G	NP	2.7–3.7	2.7–2.8	3.6–3.7	
**Globulin (g/dL)**	Males	93	2.2	0.2	2.2	1.7	2.6	0.003	NG	NP	1.7–2.5	1.7–1.9	2.5–2.6	0.0363^a^
	Females	52	2.2	0.2	2.2	1.9	2.9	0.001	NG	NP	1.9–2.8	1.9–2.0	2.6–2.9	
**A/G Ratio**	Males	93	1.45	0.14	1.43	1.04	1.80	0.003	NG	NP	1.19–1.76	1.04–1.29	1.70–1.80	0.5888
	Females	52	1.46	0.11	1.49	1.20	1.65	0.038	NG	NP	1.20–1.64	1.20–1.24	1.61–1.65	
**Cholesterol (mg/dL)**	Males	92	36	13	35	17	86	0.000	NG	NP	18–77	17–20	55–86	0.0317^a^
	Females	52	46	25	38	12	119	0.000	NG	NP	13–117	12–17	95–119	
**CK (IU/L)**	Males	89	561	435	444	107	1975	0.000	NG	NP	116–1924	107–128	1523–1975	0.7809
	Females	49	524	374	387	125	1620	0.000	NG	NP	130–1591	125–200	1295–1620	
**Total Bilirubin (mg/dL)**	Males	92	0	0	0	0	0	-	-	NP	0–0	0–0	0–0	> 0.9999
	Females	52	0	0	0	0	0	-	-	NP	0–0	0–0	0–0	
**ALP (IU/L)**	Males	93	74	32	68	29	170	0.000	NG	NP	32–160	29–36	155–170	0.0873
	Females	52	88	42	78	37	205	0.001	NG	NP	37–197	37–40	163–205	
**ALT (IU/L)**	Males	91	39	10	37	17	72	0.005	NG	NP	23–69	17–25	58–72	0.0196^a^
	Females	51	35	12	34	15	77	0.008	NG	NP	15–75	15–19	59–77	
**AST (IU/L)**	Males	91	62	30	53	22	162	0.000	NG	NP	24–142	22–27	118–162	0.1031
	Females	50	75	44	56	33	215	0.000	NG	NP	33–210	33–35	169–215	
**GGT (IU/L)**	Males	93	18	8	18	6	37	0.016	NG	NP	6–37	6–8	32–37	0.3739
	Females	52	17	7	17	0	33	0.292	G	NP	2–32	0–8	28–33	
**Sodium (mEQ/L)**	Males	93	136	3	137	127	144	0.001	NG	NP	128–132	127–132	140–144	0.4988
	Females	52	136	3	137	127	142	0.030	NG	NP	128–141	127–131	140–142	
**Potassium (mEQ/L)**	Males	92	4.93	0.79	4.81	3.66	7.21	0.000	NG	NP	3.85–6.99	3.66–3.98	6.59–7.21	0.0407^a^
	Females	52	5.37	1.16	5.00	3.89	9.90	0.003	NG	NP	3.91–9.09	3.89–4.03	6.99–9.00	
**Chloride (mEQ/L)**	Males	92	100.7	2.9	101.0	91.8	107.3	0.381	G	NP	94.1–107.2	91.8–96.1	104.9–107.3	0.1745
	Females	52	101.1	3.6	101.5	90.5	107.2	0.011	NG	NP	90.9–107.2	90.5–93.8	105.6–107.2	
**Bicarbonate (mEQ/L)**	Males	93	25.0	2.4	25.0	19.5	31.3	0.876	G	NP	19.9–29.6	19.5–21.3	28.9–31.3	0.0001^c^
	Females	51	23.4	2.3	23.6	18.5	28.4	0.530	G	NP	18.5–28.2	18.5–19.5	26.5–28.4	
**Anion Gap (mmol/L)**	Males	93	16	2	15	12	24	0.000	NG	NP	12–22	12–13	21–24	0.0102^a^
	Females	51	17	3	17	13	26	0.002	NG	NP	13–25	13–14	21–26	
**Calculated Osmolality (mOsm/kg)**	Males	93	284	5	284	269	302	0.017	NG	NP	272–293	269–273	292–302	0.0489^a^
	Females	51	285	5	285	276	297	0.801	G	NP	276–296	276–278	294–297	
**Iron (μg/dL)**	Males	70	285	30	288	197	350	0.128	G	NP	210–345	197–236	331–350	< 0.0001^d^
	Females	52	319	46	325	186	405	0.282	G	NP	199–405	186–245	390–405	

^a^*P* < 0.05

^b^*P* < 0.01

^c^*P* < 0.001

^d^*P* < 0.0001 between values for adult male and female Hartley guinea pigs. Abbreviations: G, Gaussian; NG, non-Gaussian; NP, nonparametric; RI, reference interval.

Sex-associated differences in biochemistry parameters are shown in Table 6. Males had higher ALT (median difference 3 IU/L; 95% CI 1–8; P = 0.0196) and bicarbonate (mean difference 1.6 mEQ/L; 95% CI 0.8–2.5; P = 0.0001). Females had higher total protein (mean difference 0.2 g/dL; 95% CI 0.1–0.3; P = 0.0002), albumin (mean difference 0.2 g/dL; 95% CI 0.1–0.2; P < 0.0001), globulin (mean difference 0.0 g/dL; 95% CI 0.0–0.1; P = 0.0363), cholesterol (median difference 3 mg/dL; 95% CI 0–11; P = 0.0317), potassium (median difference 0.19 mEQ/L; 95% CI 0.02–0.63; P = 0.0407), anion gap (median difference 2 mmol/L; 95% CI 0–2; P = 0.0102), calculated osmolality (mean difference 1 mOsm/kg; 95% CI 0–4; P = 0.0489), and iron (mean difference 34 μg/dL; 95% CI 21–48; P < 0.0001).

## Discussion

As comprehensive reference intervals for blood parameters have not previously been published for the Dunkin Hartley guinea pig, the purpose of this study was to establish reference intervals for hematologic and serum biochemical parameters of this strain according to the ASVCP guidelines. Age- and sex-associated differences were also determined. These results provide the foundation for interpreting hematology and serum biochemistry values of the Dunkin Hartley guinea pig.

There were several age- and sex-related changes in hematology and biochemical parameters in the Dunkin Hartley strain. However, it is important to use clinical judgement when evaluating these differences, as a statistically significant difference does not always correspond to a clinically significant difference. For example, Hct was positively correlated with age in males, leading to significantly higher levels in adults compared to juveniles. However, the median difference between juveniles and adults was 1%, and the upper limit of the reference interval was only 1% higher in adults compared to juveniles. This data will prove useful in study design and analysis when the primary endpoint is statistical detection of differences and likely will not have a significant impact on clinical interpretation of the data from a single individual.

Of the RBC parameters, males had significantly higher Hgb, Hct, RBC, and MCHC compared to females. Similar findings have been reported in numerous animal species, including other rodents [6,10,11] as well as humans [16]. This may be due to the varying effects of estrogen and testosterone on erythropoietin production [16]. These sex differences were small and unlikely to affect clinical interpretation. Although RDW was higher in females, there was a weak correlation with age in males. MCV was strongly correlated with age in females, leading to higher levels in females compared to males. Juveniles had significantly higher numbers of platelets, but lower MPV, than adults. While MPV was strongly correlated with age in both males and females, the number of platelets moderately decreased with age in males. Both platelets and MPV were significantly higher in females compared to males. In contrast, platelets were positively correlated with age in male Weiser-Maples guinea pigs [5] and C57BL/6J mice [9], and no sex differences were observed in platelets of Strain 13 guinea pigs [6]. Platelets in Sprague-Dawley rats were shown to markedly decrease with age, and later increase in old age [12]. Additionally, platelet numbers were higher in male 129SV/EV and C3H/HeJ, but not C57BL/6J, mice compared to females [9]. Clinical relevance of this variability in platelet numbers among different sexes and strains of rodents is unknown and may be worthy of additional research.

Similar to other strains of guinea pigs [5,6], juvenile guinea pigs had higher numbers of heterophils and lower numbers of lymphocytes compared to adults. In both sexes, heterophils markedly increased with age, while lymphocytes decreased with age. Additionally, total WBC, Foa-Kurloff cells, monocytes, and eosinophils mildly increased with age and Foa-Kurloff cells markedly increased with age in males, but not females. Although females had more eosinophils, males had significantly higher WBC counts, due to higher numbers of heterophils and Foa-Kurloff cells. Foa-Kurloff cells are a type of estradiol-dependent white blood cell unique to guinea pigs that contain a large granular intracytoplasmic inclusion. Although the function of these cells is unknown, they are thought to have natural killer cell activity [17] and protect the fetus during pregnancy. Foa-Kurloff cells are commonly associated with pregnancy in older females and are reported to rarely be seen in young animals or males [18]. In contrast, higher numbers of Foa-Kurloff cells were seen in males compared to females in the current study. Additionally, higher numbers were reported in male strain 13 guinea pigs than females [6]. Other studies did not report numbers of Foa-Kurloff cells [4,5]. As this cell type is not recognized in automated hematology analyzers, manual differential leukocyte counts are particularly important for accurate white blood cell counts in guinea pigs.

Glucose was positively correlated with age in male Dunkin Hartley guinea pigs, but negatively correlated in strain 13 males. Additionally, our reference intervals for glucose were much higher than those reported for strain 13 and Weiser-Maples guinea pigs [5,6], which may be due to variations in diet or fasting status. Animals were not fasted prior to blood collection in the current study. As fasting has been shown to affect numerous clinical pathology parameters in rats [19], it may also influence similar parameters in guinea pigs.

Like other guinea pig strains, BUN, creatinine, and calcium were positively correlated with age [5,6]. An increase in these parameters may be associated with the development of renal disease. Spontaneous renal lesions, such as nephrosclerosis, are a common incidental finding in guinea pigs that may result in renal insufficiency. Total protein, albumin, and globulin were also significantly increased with age in both sexes, with females having higher levels compared to males. A similar age-related increase in total protein was observed in Weiser-Maples guinea pigs [5], but not strain 13 guinea pigs [6]. Although total bilirubin measured 0 mg/dL in all guinea pigs included in this study, this value is consistent with reference intervals of other guinea pigs [18].

ALP and phosphorus significantly decreased with age, likely due to the decline in bone growth as animals reached skeletal maturity. In the guinea pig, bone growth is purported to cease by 4 months of age [20]. Liver enzymes ALT and GGT markedly increased with age in males, and AST increased with age in both sexes. However, these enzymes were not correlated with age in other strains [5,6]. Male Hartleys had significantly higher levels of ALT than females, whereas strain 13 males had significantly higher levels of ALT, AST, and GGT than females. Increased levels of these enzymes may be indicative of hepatocellular and/or biliary disease.

Consistent with Weiser-Maples guinea pigs [5] and Sprague Dawley rats [10,12], cholesterol increased with age in males, but females had higher overall levels compared to males [5]. Dunkin Hartley females also had an age-associated increase in iron, with significantly higher levels compared to males. In contrast, iron decreased with age in both male and female mice [9]. Magnesium was positively correlated with age in males, leading to significantly higher levels in adults than juveniles. Male guinea pigs had higher levels of bicarbonate, whereas females had higher potassium, anion gap, and calculated osmolality. Additionally, bicarbonate was positively correlated with age and potassium was negatively correlated with age in males, which led to a negative age correlation in anion gap. Similar to Weiser-Maples guinea pigs [5], sodium was positively correlated with age in female Dunkin Hartley guinea pigs. Iron, magnesium, bicarbonate, and calculated osmolality were not evaluated in other guinea pig studies. The reason for these age- and sex-related differences in electrolytes is unknown and warrants further investigation.

In addition to strain, age, and sex, blood parameters may be affected by other factors that should be considered when applying these reference intervals to other animals. For example, the majority of guinea pigs included in this study were anesthetized with isoflurane for blood collection. As blood collection is challenging in this species, anesthesia is often necessary to collect large blood volumes to minimize trauma and stressful handling. Isoflurane has been shown to increase white blood cells and liver enzymes and decrease red blood cells and plasma proteins in Dunkin Hartley guinea pigs [21]. Additionally, this study included data from blood collected from the cranial vena cava and jugular vein, as well as directly from the heart. In rodents, hematologic and biochemical parameters can vary when blood is collected from different anatomical locations [22–27]. It should also be noted that all guinea pigs in this study were housed in Colorado at an elevation of approximately 5,000 ft, which may impact certain RBC parameters. For example, humans at high altitude have increased erythropoietin and hemoglobin levels, as well as increased red blood cell volume [28]. Future work would assess a comparison of subjects at high altitude versus sea level.

In conclusion, this study found several important differences in hematologic and biochemical parameters of Dunkin Hartley guinea pigs based on age and sex. Additionally, our results showed many differences between Dunkin Hartley and other strains of guinea pigs, which further emphasizes the need for strain-specific reference intervals. Establishing these differences provides valuable insight into their physiology to better evaluate diagnostics and experimental results.
